# Hepatocellular Carcinoma in Non-Fibrotic Liver: A Narrative Review

**DOI:** 10.3390/diagnostics13223426

**Published:** 2023-11-10

**Authors:** Hiroko Naganuma, Hideaki Ishida

**Affiliations:** 1 Department of Gastroenterology, Yokote Municipal Hospital, Negishi-cho 5-31, Yokote City 013-8602, Japan; 2 Department of Gastroenterology, Akita Red Cross Hospital, Kamikitate Saruta aza Naeshirosawa 222-1, Akita City 010-1495, Japan

**Keywords:** hepatocellular carcinoma, non-fibrotic liver, tumor capsule, AFP, risk factor, hypervascular tumor

## Abstract

Hepatocellular carcinoma (HCC) in a non-fibrotic liver (F0) is considered to be rare, and there is a marked paucity of studies in the literature on this HCC type. A review of the literature shows some important clinical and tumor characteristics: (a) it occurs mainly in young female and elder male patients; (b) clinically, under normal hepatic function, alpha-fetoprotein level is often normal, and there are no risk factors; (c) associated with metabolic disease; (d) macroscopically, single large lesions are noted; and (e) microscopically, the lesions are well-differentiated and encapsulated. Radiological imaging results are straightforward, showing arterial hyperenhancement and later wash-out. The combined use of B-mode and contrast-enhanced (CE) ultrasound (US) is the most reliable and cost-effective diagnostic method. Few peri-and post-operative complications are noted and 5-year survival is not inferior to patients with HCC on fibrosis liver despite the lesion’s large size. Most clinicians believe that HCC is unlikely to occur if patients have no symptoms and normal hepatic function. Although detailed clinical data are very limited, we expect that this review will help to improve the clinical management of HCC in non-fibrotic livers.

## 1. Introduction

Hepatocellular carcinoma (HCC) ranks fifth in cancer incidence and third in cancer mortality worldwide [1,2]. The development of HCC is complex, but it typically arises in the liver, with chronic changes [3]. The major risk factors reported in the literature include chronic hepatitis viral infection, cirrhosis, and alcohol consumption [4,5]. In addition to these classical risk factors, many recent clinical studies have shown non-alcoholic steatohepatitis (NASH) [6,7]. Diabetes mellitus, metabolic syndrome, and obesity [8] are implicated in HCC development. Other, less common, risk factors for developing HCCs include aflatoxin exposure, tobacco smoking, hemochromatosis, alpha-antitrypsin deficiency, and porphyria cutanea tarda [8]. There are a number of reports on HCCs in non-cirrhotic livers [9,10], but their non-cirrhotic liver groups consist of patients with very heterogeneous degrees of fibrosis (from F0 to F3) [10,11,12,13,14], and this heterogeneity prevents us from grasping the precise images of HCC in the non-fibrotic (F0) liver, although some of these reported non-cirrhotic liver cases would have naturally included a number of F0 liver cases. The liver fibrosis stage was determined by using a staging system consisting of five stages (stage 0, healthy liver without fibrosis (F0); stage 1, fibrosis of portal area without septa (F1); stage 2, portal fibrosis with few septa (F2); stage 3, septal fibrosis without cirrhosis (F3); stage 4, cirrhosis (F4)) [15]. The application of this staging system is necessary, because in a significant number of reported cases with HCC in “non-fibrotic liver”, the authors included many F1–F2 fibrosis cases without distinction, mainly on the idea that there would not be much difference between them [12,13,14,16,17,18]. In other words, most of these studies have missed valuable opportunities to clarify the real image of HCC arising from non-fibrotic liver [15]. Thus, the non-fibrotic liver is strictly defined as a liver with no fibrosis (F0). As a result, some important questions are raised: (1) are there no particular clinical characteristics in patients with HCC in a non-fibrotic liver (F0)?; (2) what predisposing factors do they have? The aim of this narrative review is to summarize the existing data, although very limited, on HCC in a non-fibrotic liver (F0) with special attention to the clinicopathological characteristics, treatment, and outcomes of patients with this rare HCC type to answer these questions.

## 2. Incidence of HCC in Non-Fibrotic Liver (F0)

**Figure 1:**
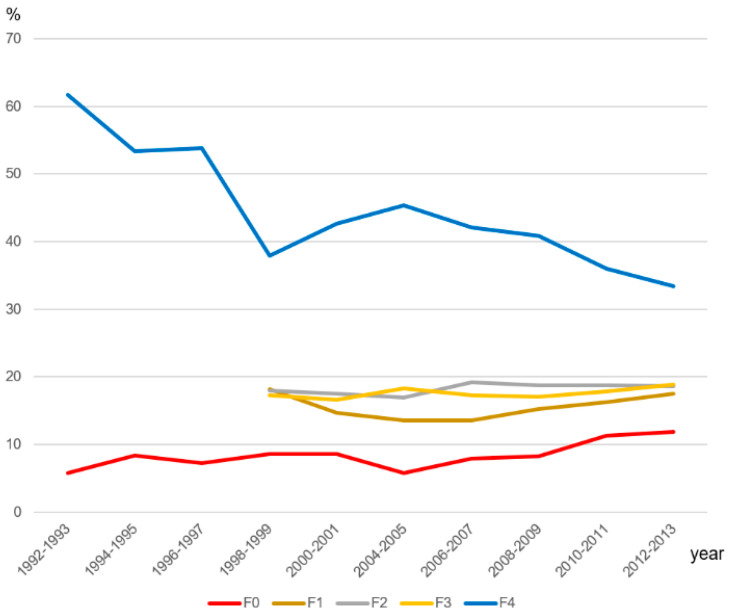
Incidence of F0 (non-fibrotic liver), F1, F2, F3, and F4 (cirrhosis) cases among all Japanese HCC cases. The graph shows change in incidence of background fibrosis in the liver in all HCC cases based on a series of annual reports from a Japanese multicenter survey. In 1992, HCC in non-fibrotic livers accounted for approximately 5.8% of resected HCCs in Japan; this number has grown each year and reached 10.8% in 2012. This curve also shows a rising incidence of HCC in chronic hepatitis (F1–F3) along with a parallel gradual increase in non-fibrotic livers (F0) with inversely decreasing incidence of liver cirrhosis.

The analysis in this review essentially targets human studies in English included in an annual report on HCC from the Follow-up Survey Committee of the Liver Cancer Study Group of Japan, as it is the sole source of cumulative data on HCC in the world. This series of reports deals with a Japanese multicenter survey of HCC. In 1992, HCC in non-fibrotic livers accounted for approximately 5.8% of resected HCCs in Japan, and this number gradually increased every year, reaching 10.8% in 2012 [19,20,21,22,23,24,25,26,27,28,29,30,31]. This survey also clearly shows a rising incidence of HCC in non-fibrotic livers, along with a parallel gradual increase in chronic hepatitis (F1–F3), with an inversely decreasing incidence of LC (Figure 1). This tendency suggests that in the coming years, the incidence of HCC in non-fibrotic livers will continue to increase. However, this huge Japanese database focuses mainly on histological classification and lacks details on the majority of cases of HCC in non-fibrotic livers, and the etiology and clinical characteristics remain unclear in this annual report. Many data have been sporadically published from various geographic regions and populations [9,10,32,33,34,35,36,37], but these studies have not been described in detail (e.g., fibrosis staging). Strictly speaking, the applicability of the Japanese data must be confirmed in the future through many follow-up studies from various geographic regions and populations.

## 3. Materials and Methods (Literature Search and Study Selection)

Search strategy and data abstraction: The literature search aimed to identify all relevant entries in the literature regarding hepatocellular carcinoma in the non-fibrotic liver. The PubMed and Medline databases were systematically searched for relevant articles published between Jan 2000 and May 2023 using the terms “hepatocellular carcinoma”, “hepatoma”, “primary liver tumor”, “liver cancer” ×“normal liver”, “non-fibrotic liver”, “healthy liver”, and “liver fibrosis”. Additional search methods included a manual review of the reference lists of relevant studies. The inclusion criteria were as follows: (1) the study was published with extractable clinical information, including sufficient medical imaging results, (2) the tumor was surgically resected and histologically confirmed, and (3) the surrounding liver parenchyma was carefully examined and the final histological diagnosis of an F0 liver was established. Abstracts or posters were not selected because their quality was difficult to evaluate. Studies that grouped F0 and F1 livers together as minimal fibrosis or no significant fibrosis were not included because this review focused on F0 livers only. Four child studies were not included because we reviewed adult HCC in non-fibrotic liver cases only in this review. Two reviewers (HN and HI) independently examined the titles and abstracts of the publications according to the search strategy. In this review, only articles published after January 2000 were selected, since articles published before this year provided insufficient data regarding clinical findings and medical imaging results.

## 4. Results

**Figure 2:**
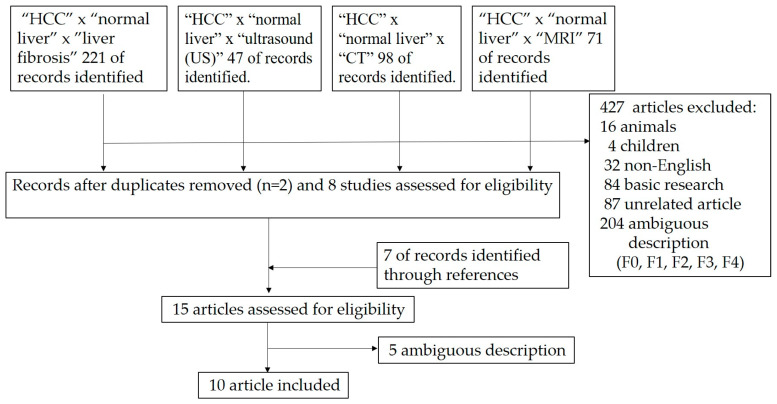
Study selection schema.

Initially, 437 articles were retrieved from our review. Additionally, seven articles were obtained through the manual review of the reference lists of relevant articles. From these 444 articles, 427 were excluded because of the following reasons: (1) 204 articles grouped F0 and F1 livers together and (2) 223 articles had insufficient clinical data. Finally, 10 articles were selected (Figure 2).

Our review of these 10 reports found the following clinical and tumor characteristics of HCC in non-fibrotic livers:

### 4.1. Patient Demographics and Clinical Data

Our final review of HCC in the non-fibrotic liver identified 124 patients. The clinical data are shown in Table 1 [38,39,40,41,42,43,44,45,46,47]. They show a slight male preponderance (66% (M) vs. 34% (F)) ranging in age between 23 and 86 with a mean of 59.8 years old. In seven of these ten articles, the patient’s demographics were described in detail [40,41,42,43,45,46,47]. According to these reports, the 10 female patients had a mean age of 56.9 years old, ranging between 23 and 83. Five of the ten females were less than 50 years old. The 10 male patients had a mean age of 71.0 years old, ranging between 60 and 86. Eight of the ten males were more than 65 years old. Thus, they were bi-modal in age distribution (a small peak of relatively young females and a large peak of elder males). No patient received follow-up surveillance using medical imaging. There were a very small number of patients with hepatitis viral infection, hepatitis C in 4/124 (3.2%) and hepatitis B in 13/124 (10.4%). There was normal hepatic function in 13/19 (68.5%) patients, slight hepatic dysfunction in 6/19 (31.5%) patients, and marked hepatic dysfunction in 0 patients [40,41,43,45,46,47]. The serum AFP level was normal in 56/97 (57.7%) patients [39,40,41,42,43,44,45,46,47]. The symptoms included abdominal pain in 2/20 (10%) patients, general fatigue in 1/20 (5%) patients, back pain in 1/20 (5%), abdominal mass in 1/20 (5%), and no symptoms in 15/20 (75%); additionally, no patients showed some severe symptoms [40,41,43,45,46,47]. There were 6/19 (31%) patients of metabolic disease (five DM cases, one hyperlipidemia case). The presence or absence of multiple primary malignancy (MPM) were usually not described, except for Naganuma’s report [45]. According to this report, three of twelve cases were MPM-associated (25%).

### 4.2. Tumor Characteristics and Medical Imaging Findings

The tumor characteristics and medical imaging findings are also shown in Table 2, although the description of each finding was sometimes ambiguous [38,39,40,41,42,43,44,45,46,47]. All patients had relatively large tumor sizes (mean: 94.5 mm, (ranging from less than 20 to 250 mm)) [38,39,40,41,42,43,44,45,46,47]. The HCC lesion was singular in 92/114 (80.7%) cases and multiple in 22/114 (19.3%) cases [38,39,40,41,42,43,45,46,47]. Histologically, the HCC lesion was microscopically well-differentiated in 8/57 (14.0%) patients, moderately differentiated in 19/57 (33.3%) patients, well-moderately differentiated in 19/57 (33.3%) patients, poorly differentiated in 5/57 (8.7%) patients, and unknown in 6/57 (10.5%) patients [38,40,41,42,43,44,45,46,47]. The lesion was encapsulated in 96/120 (80%) patients and non-encapsulated in 24/120 (20%) patients [38,39,41,43,44,45,46,47].

On medical imaging, the HCC lesion showed arterial hyperenhancement in 26/27 (96%) patients and hypoenhancement in 1/27 (4%) patients in the arterial phase; additionally [41,42,43,44,45,46,47], there was wash-out in 20/26 (77%) patients and no wash-out in 6/26 (23%) patients in the later phase [41,42,44,45,46,47]. Tumor vascularity and tumor capsule were evaluated mainly via CT and MRI 13/26 (50%) and via CT, MRI, and CEUS in 13/26 (50%). B-mode sonography showed the tumor capsule, including lateral shadowing suggestive of the presence of a tumor capsule, for 12/13 (92.3%) patients.

### 4.3. Treatment and Outcome

In total, 124 patients underwent tumor rection and no patients received liver transplantation. Liver resections were performed by open approach according to the surgeon’s usual practice with their own parenchymal transection equipment, yielding a low operative mortality rate (0–3.7%) [38,45]. A long (more than 5 years) follow up after HCC resection was reported in only one series [38]. According to this report, the overall five-year survival and disease-free survival in the non-fibrotic group (27 patients) was slightly better than HCC on diseased liver (141 patients), yielding 87.5% and 41.7% (over-all five-year survival and disease-free five-year survival) in the non-fibrotic group vs. 62.3% and 33.7% in the fibrotic liver group. In this report, tumor recurrence occurred in 67% in the non-fibrotic group vs. 60% in the fibrotic liver [38].

## 5. Reported Possible Causal Factors of HCC in Non-Fibrotic Liver (F0)

When investigating HCC arising from a normal liver, the following contributing factors must be considered.

### 5.1. Family History and Genetic Factors

Familial aggregation of HCC has been reported without any clear suggestions of an underlying genetic predisposition [48,49,50]. However, these clinical studies did not provide any detailed information [48,49,50]. To date, no studies have shown a strict association between family history and genetic factors and the risk of developing HCC in a non-fibrotic liver [41,45].

### 5.2. Aging and Sex

As has been investigated by many studies, aging is characterized by a progressive deterioration of all physiological functions [51]. These changes, together with an increase in accumulating risk factors, predispose individuals to develop many cancers [52,53]. Thus, elders are thought to have an increased risk of developing HCC [54,55]. This idea can be applied to HCCs in non-fibrotic livers. In fact, according to a relatively large sample case series [45], the average age of patients who present with HCC in a non-fibrotic liver at initial diagnosis is more than 70, which is approximately 10 years older than the average age of patients with HCC (mainly with liver cirrhosis) [56], emphasizing the association between aging and HCC. As regards sex, Naganuma’s series of twelve patients consisted of eight men and four women. Thus, aging and sex are possible contributing factors to the development of HCC in a non-fibrotic liver.

### 5.3. Alcohol Consumption

A strong association between alcohol abuse and HCC development has been emphasized [57], but mainly through the formation of liver cirrhosis [58]. The effects of alcohol consumption on the development of HCC are considered to be stronger with advancing age, but usually through the formation of liver cirrhosis [58]. Except for Takahashi’s case study [41], in the other cases of HCC in non-fibrotic livers, the patients consumed almost no alcohol. Thus, alcohol is not thought to be a contributing factor to HCC in a non-fibrotic liver.

### 5.4. Obesity and Diabetes Mellitus (DM)

In obese patients, HCC usually appears as a late consequence of long-standing metabolically associated fatty liver disease (MAFLD) [59]. MAFLD refers to liver steatosis in addition to obesity, overweight, DM type2, and/or metabolic dysregulation [59]. Patients with MAFLD-related HCC have been reported to be older than patients with HCC and other underlying diseases (73 years vs. 66 years) and to have a shorter survival time after the occurrence of HCC [60]. In patients with obesity and/or DM, HCC generally appears in fatty liver with fibrosis. In a case series of HCC in non-fibrotic livers, three of twelve cases (25%) were associated with DM, but all of them were well-controlled with drug therapy, and DM was likely to be a causal factor also in HCC in non-fibrotic liver [45].

### 5.5. Viral Infection

Infection with hepatitis B or C is the most common etiology of HCC, occurring in more than 50% of all HCC patients [61,62,63]. However, this association was found to be extremely uncommon in patients with HCC in a non-fibrotic liver [38,39,40,41,42,43,45,46,47]. As a result, viral hepatitis infection was not considered to be a causal factor of HCC in non-fibrotic livers.

### 5.6. Autoimmune Hepatitis

Autoimmune liver diseases, including autoimmune hepatitis, primary biliary cholangitis, and primary sclerosing cholangitis, are well-known pathologies that occur mainly in women and are associated with HCC in some cases [64,65]. However, no cases have been reported in patients with non-fibrotic livers associated with autoimmune diseases.

### 5.7. Exposure to Chemicals

Exposure to chemicals, including polyvinyl chloride and arsenic, is known to induce hepatic malignancies [66]. However, hepatic angiosarcoma represents the majority of chemical-induced hepatic tumors, and the occurrence of HCC is not common.

### 5.8. Multiple Primary Malignancies (MPMs)

The association of multiple primary malignancies (MPMs) and HCC has rarely been reported [67,68,69], but this incidence is probably underestimated. The criteria for MPMs are as follows: (1) each tumor must present a definite picture of malignancy and (2) must be histologically distinct, and (3) one tumor being a metastasis of another must be excluded [67,68,69]. The incidence of MPM-related HCC was thought to be approximately 2–5%. In Naganuma’s series, three of thirteen cases (23%) were MPM-related, much higher than usually expected. Although this series had a small sample size, this phenomenon must be taken into consideration when caring for patients with HCC in non-fibrotic liver.

### 5.9. Hepatocellular Adenoma (HCA)

HCA comprises a group of rare benign tumors that occur predominantly in women who use oral contraception, but also in patients who have diabetes mellitus, use androgens, or have glycogen storage disease [70]. HCA is divided into four subtypes: beta-catenin, inflammatory, HNF1alpha-mutated, and unclassified. HCA is basically a benign tumor but has malignant potential. The risk of malignant transformation has been reported to be 4–5% in patients with hepatocellular adenoma [71], and the risk factors for malignant transformation include the size (>5 cm), male gender, and an activating mutation in β-catenin [72].

### 5.10. The Other Less Common Risk Factors

These include aflatoxin exposure, tobacco smoking, hemochromatosis, alpha- antitrypsin deficiency, and porphyria cutanea tarda [8]. However, the reported cases of HCC in non-fibrotic livers were not associated with these factors. They also included Abernethy malformation, Alagille syndrome, ataxia telangioectasia, bile salt protein deficiency, tyrosinemia type 1, hypercitrullinemia, fructosemia, and others in children HCC [73].

The histopathogenic pathways of the evolution of HCC arising from non-fibrotic hepatic parenchyma remain difficult to determine due to the lack of fibrosis. One of the most likely possibilities is that an HCC focus may be generated from precursor cells derived from the existing adenomatous tissues. The origin of HCC tissue is an important problem to be resolved in the near future.

Additionally, the problems of aging and MPMs are not negligible. Aging has been considered as a major risk factor for developing cancer through some underlying mechanisms including long-standing DNA damage, cellular senescence, and immunosenescence [65,66,67,68,69,74,75]. On the other hand, cancer patients have received increasing survival benefits from continuous progress in early cancer detection and intensive treatments. Along with increased life expectancy, cancer survivors are at high risk of developing another cancer compared with the general population [67,68,69].

## 6. Clinical Manifestation and Diagnosis

**Figure 3:**
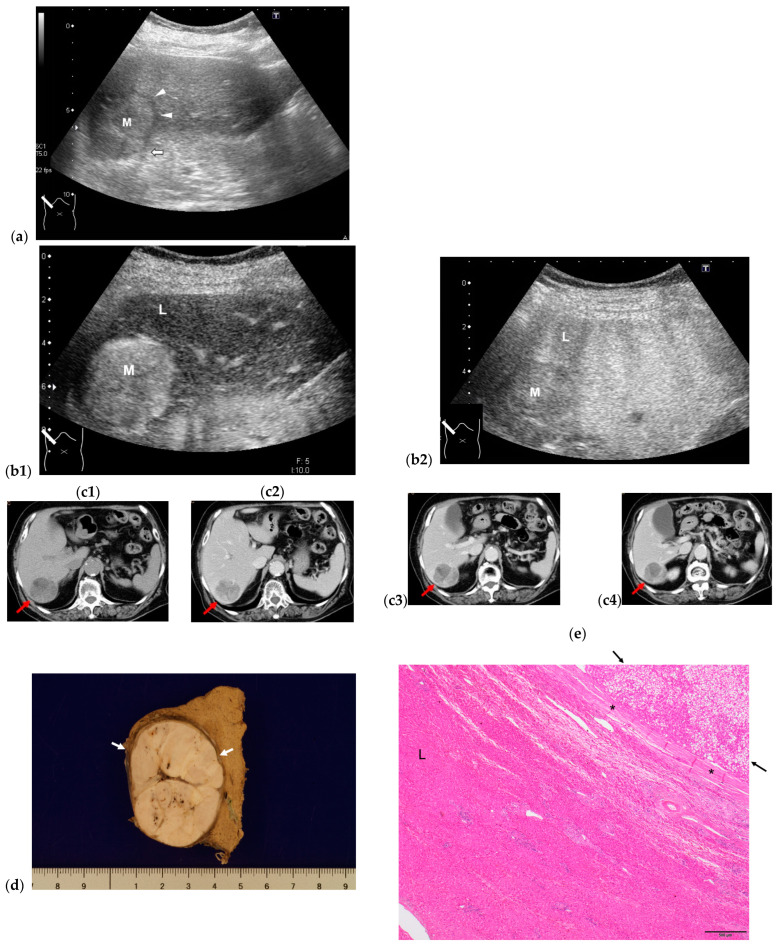
Representative case of HCC in non-fibrotic liver (F0). (**a**) A large 4.6 cm× 3.5 cm hypoechoic round mass (M) with halo (arrowheads) and lateral shadowing (arrow) is seen on B-mode US. L: liver. CEUS clearly shows arterial hyperenhancement (**b1**), with iso-enhancement in the portal venous phase, and late wash-out (**b2**). CT findings reveals a solitary mass in S7 in pre-contrast scanning (arrow) (**c1**). Lesion shows arterial hyperenhancement (**c2**) and late wash-out (**c3**) portal venous phase, and (**c4**) equilibrium phase. (**d**) Macroscopically, lesion (arrows) is well-demarcated in non-fibrotic liver. (**e**) Microscopically, lesion mainly consists of well-differentiated HCC (arrows). Surrounding liver parenchyma (L) appears to be normal. *: capsule. This patient is symptom-and recurrence-free 7 years after HCC resection.

Cho et al. [76] and Nishikawa et al. [77] have noted some clinical and tumor characteristics seen in HCC in elderly patients: (a) clinically, a lack of surveillance and no risk factors; (b) macroscopically, a single large lesion; and (c) microscopically, a well-differentiated encapsulated lesion without vascular invasion. Although these HCC cases were associated with a fibrotic liver, their tumor characteristics were also seen in HCC arising from a non-fibrotic liver [45]. The radiological findings faithfully represented these histologic characteristics, and the radiological diagnosis was straightforward according to Liver Imaging and Data System (LI-RADS) category 5 (definitely HCC), consisting of arterial phase hyperenhancement and late-phase wash-out [78,79]. Although recent meta-analyses have shown that contrast-enhanced ultrasound (CEUS) has higher sensitivity than CT or MRI for the diagnosis of HCC in chronic hepatic disease, there is currently insufficient evidence to strongly recommend CEUS for HCC in a non-fibrotic liver over the other two modalities, and further cases are necessary to draw a definitive conclusion. Homogeneous arterial enhancement on CEUS is worth noting. The most likely explanation for this enhanced pattern is the absence of a poorly differentiated HCC component in HCC arising from a non-fibrotic liver, despite its large size (Figure 3).

## 7. Differential Diagnosis

Strictly speaking, any large solitary hypervascular mass in a non-fibrotic liver must be included in the differential diagnosis. We must consider the pathologies listed below. Most of these lesions are asymptomatic, with tumor markers found to be within the normal range. Thus, accurate radiologic evaluation is critical.

### 7.1. Fibrolamellar Hepatocellular Carcinoma (FL-HCC)

FL-HCC accounts for less than 1% of all primary malignant tumors of the liver [80]. These tumors occur predominantly in young patients without underlying liver disease [80]. FL-HCC lesions are usually large, single, and well-defined, but they usually contain a calcified central scar, unlike HCC in non-fibrotic livers. FL-HCC lesions show hypervascularity in the arterial phase and isovascularity in the venous phase. No lateral shadowing around FL-HCC lesions is seen on US.

### 7.2. Hepatocellular Adenoma

It is usually very challenging to distinguish HCA from well-differentiated HCC, mainly due to the lack of a unified definition and variability in interpretation among pathologists, especially of biopsy specimens [81]. HCA usually shows marked hypervascularity and/or slow, faint wash-out, mimicking HCC in a non-fibrotic liver. Thus, at present, it is almost impossible to differentiate HCC in a non-fibrotic liver from HCA via medical imaging or small biopsy specimens. Thus, a resection of the lesion and detailed interpretation of the surgical specimen are indispensable for establishing a definitive diagnosis of HCC in non-fibrotic livers [45].

### 7.3. Focal Nodular Hyperplasia (FNH)

CEUS plays a crucial role in differentiating FNH from other liver tumors. FNH presents the following characteristic findings: (a) hyperenhancement of the whole lesion, with fast centrifugal filling of the lesion, composed of central vessels and radial vascular branches at the early arterial phase (so-called “spoke-wheel appearance”) and (b) iso-enhancement of the whole lesion in the portal phase and afterward [82,83]. The visualization or non-visualization of a central scar in the delayed phase is usually not included in the diagnostic criteria because the visualization of an avascular area largely depends on the size of the central scar, and clear visualization of the central scar is thought to be possible less than 50% of the time [83,84]. In a word, these CEUS findings lead to the final diagnosis of FNH, regardless of the lesion size.

### 7.4. Hypervascular Hemangioma (Hy-Heman)

It has long been well known that a small number of hepatic hemangiomas are markedly hypervascular, mainly due to developed arterioportal shunts [85]. In the past, clinicians had difficulty differentiating hemangiomas of this type from other hypervascular tumors. However, since the introduction of CEUS in the clinical setting as a detailed diagnostic tool for liver tumors, this differentiation is not so difficult. CEUS reveals not only the hypervascularity of lesions due to arterioportal shunts, but also a “cotton-wool” appearance in the periphery, leading to the diagnosis of hypervascular hemangioma [86].

### 7.5. Solitary Metastasis

Colon cancer and sarcoma are frequently associated with solitary hypervascular liver metastasis, mimicking HCC [87]. However, colon cancer metastasis usually involves many calcifications, and this B-mode finding helps in diagnosing lesions as liver metastasis from colon cancer [88]. In addition, a high serum level increases the likelihood of diagnosing hypervascular solitary metastasis, such as CEA.

**Figure 4:**
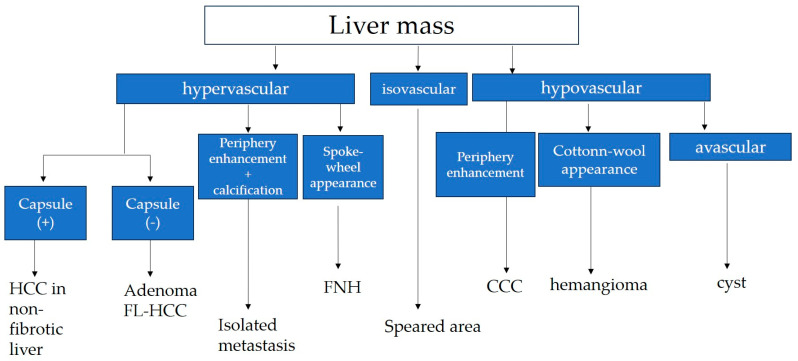
Diagnostic algorithm of liver mass in normal hepatic function. HCC: hepatocellular carcinoma; FL-HCC: fibrolamellar HCC; FNH: focal nodular hyperplasia; CCC: cholangiocellular carcinoma.

The most important point in the differential diagnosis of HCC in non-fibrotic liver from other hypervascular tumors is the presence of a capsule, which is imaged most clearly by B-mode US as lateral shadowing, which is a phenomenon caused by the refraction of US beams when passing through the capsule [89]. A diagnostic algorithm for general clinicians is shown in Figure 4.

## 8. Treatment and Outcome

Patients with HCC in a non-fibrotic liver present late, so they usually have large tumors. The well-preserved liver function allows a more extensive liver resection in these patients, making it more likely that an R0 resection will be successful (i.e., margins free of macro- and microscopic tumors). In fact, as has been shown in the literature, extensive liver resection can be performed quite safely in these patients, as evidenced by the low operative mortality rate (0–3.7%) [38,45]. Long-term follow-up studies have clearly shown that surgical resection is currently the only curative treatment for any HCC in non-fibrotic liver [41,45,46]. In short, most studies show that advanced age alone should not be a reason to dismiss any intensive HCC treatment [38,45]. However, given that the majority of patients with HCC in a non-fibrotic liver are elderly, sufficient considerations must be given to identifying which patients would most likely benefit from surgery. There may potentially be a role in the future for chemotherapy or transplantation in selected cases. The enhanced feasibility of large liver resection has shifted our attention from perioperative mortality to tumor recurrence [38,39,45]. These large series yielded almost the same results and postoperative complications were few (0–15%), despite R0 resection [38,39,45]. The overall cumulative survival rates were 87.5% and 41.7% over-all five-year survival and disease-free five-year survival, respectively [38], which are slightly better than those for HCC on diseased liver [38]. It remains unanswered what factors led to recurrence in those studies, but microvascular invasion seems to be the most likely hypothesis regarding tumor recurrence shortly after resection. Thus, the early diagnosis of tumor recurrence is considered to be especially important because it may allow repeat resection or RFA therapy with better long-term outcomes. Consequently, as has been reported for patients with HCC in a fibrotic liver [76,90], a positive attitude towards tumor resection by physicians should be justified, but close postoperative surveillance is needed. In the near future, the number of advanced and inoperable cases is expected to increase in parallel with the increase in number of detected patients of HCC in non-fibrotic livers. Thus, there may potentially be room in the future for the administration of radiofrequency ablation, microwave ablation [91], transarterial chemoembolization [92], systemic therapy [93], radiation therapy [94], and immunotherapy [95] in selected cases.

## 9. Screening System

When caring for HCC patients, the stage at cancer diagnosis is the most important factor determining outcome. Patients with small, localized tumors can undergo curative treatments such as resection or ablation [96,97]. As has been emphasized in many studies, there is a clear association between HCC surveillance and improved outcomes, and ultrasound and AFP are considered to be the standard methods [56,98,99]. Thus, most major guidelines recommend HCC surveillance for patients with chronic hepatitis [98,99,100]. However, it is not realistic to expect that HCC surveillance will be effective at improving outcomes for healthy individuals, because it generally relies not only on the accuracy of the surveillance tests and economic efficiency but also on the real-life implementation of surveillance. If annual screening of the abdomen using ultrasound is performed on all elderly patients (more than 60 years old) in the future, it will allow early detection of HCC in non-fibrotic livers, which will lead to a higher chance that treatment will be curative. However, before a screening system can be implemented, there must be serious discussion regarding the rationale and indications for HCC surveillance of elderly individuals who are not at risk. However, given the high percentage of MPM-related HCC cases [45], the most effective and realistic standard practice to detect HCC in a healthy liver is the strict follow-up of postoperative patients in daily clinical practice.

## 10. Discussion

Since the majority of HCC cases have cirrhosis in the background, fibrosis of the liver has been emphasized too much as a trigger for the development of HCC [1,2,3]. From the viewpoint of fibrosis progression, it has been elucidated that the fibrosis of viral hepatitis progresses to cirrhosis over time, which then eventually leads to the development of HCC [1,2,3]. However, this theory has delayed the studies on HCC without cirrhosis (F4) or advanced chronic hepatitis (F3). In line with these trends, the usual idea was that the F1 and F2 livers were probably in the preliminary stages of gradual transition to F3 and F4. The extreme theory that emphasizes this viewpoint is that HCC does not occur in the F0 liver. Is this notion that “HCC will not occur in the F0 liver” correct? This question was the first step in preparing this review. Along with the idea that HCC develops on advanced liver fibrosis, the common brief among general clinicians is that HCC cases are usually accompanied by liver dysfunction, hepatitis viral infection, and positive AFP. Certainly, it is relatively rare to encounter cirrhotic cases without hepatic dysfunction at all, but can the idea that HCC cases usually show liver dysfunction also be applied to HCC occurring in a non-fibrotic liver (F0)? This was another point of focus in preparing this review. In this review, we analyzed reports published after 2000, mainly because medical imaging was not widely used and the patient data were usually insufficient before 2000. Historically, the first detailed clinical report of HCC in a non-fibrotic liver was made by Okuda et al. in 1989 [11]. Their three cases of HCC in non-fibrotic livers consisted of two young men (28-and 46-year-old painter–plasterers) and a 64-year-old farmer, all without hepatitis viral infection; thus, the authors speculated that HCC in non-fibrotic livers occurred in young men and was induced by chemicals. This report was followed by a small number of clinical reports in HCC in non-fibrotic livers with the same clinical characteristics. Thus, historically, HCC in non-fibrotic livers was thought to occur in young patients for a certain period [10]. Although this historical description is very valuable, it was no more than a reference material because of the lack of diagnostic techniques and different treatment systems in those days. In brief, Okuda’s report was not thought to be a broad review of the trend at that time. Therefore, we decided to include only reports from after 2000, when the standard of medical care had improved. The most difficult point in reviewing the previous papers at the time of preparing this review was that there were only a small number of articles that dealt purely with F0 HCC or clearly separated F0, F1, and F2 HCCs [38,39,45]. Moreover, since most of them were case reports [40,41,42,43,46,47], the overall number of cases was small, and statistical analysis had not yet been conducted, leaving only a rough trend visible. However, the general trend that emerged from it was that clinically, HCC in F0 liver (1) was not associated with abnormal liver function, (2) did not show elevated AFP, (3) showed a high frequency of metabolic disease (31%), and, histologically, (4) HCC appeared as a single, large, encapsulated lesion of well-to-moderate differentiated tumor.

Therefore, HCC in F0 liver is imaged as a homogeneous hypervascular mass with wash-out on medical imaging and can be easily diagnosed using any of the current imaging techniques (US, CT, and MRI). The most important point of differentiating HCC from other hypervascular tumors is the presence of a capsule, which is imaged most clearly using B-mode US with lateral shadowing, which is a phenomenon caused by the refraction of US beams when passing through the capsule [89]. A diagnostic algorithm for general clinicians is shown in Figure 4. The rising incidence of metabolic disease-related HCC is a phenomenon worthy of note [101,102]. For example, the incidence of HCC is reported to be 0.03% in patients with NAFLD at a stage earlier than cirrhosis [103], and the degree of fibrosis is thought to be significantly associated with HCC development. However, there are no previous reports describing the association between metabolic disease and HCC in non-fibrotic liver. In this respect, the fact that 31% of HCC in non-fibrotic liver is worthy of note, and the precise pathomecanism must be resolved in the future. The important limitation with this review is that most reports in the literature were from limited regions (Japan and France) and only a small number of articles dealt purely with F0. Thus, further study is needed to determine whether these characteristic findings are generalized to the whole world.

Our review of the literature showed two peaks (a small peak of relatively young females and a large peak of elder males) in the occurrence of HCC in non-fibrotic livers, and the association between this HCC type and age is worth analyzing in the future. As shown in Incidence (Figure 1), as HCC in F0 livers accounts for close to 10% of all HCC in Japan, it is worth clarifying the pathomechanism of HCC in F0 livers. As for the most possible pathomechanism of HCC in F0 livers, it is likely to occur from adenomas. However, strictly speaking, it remains unclear but seems to be different from the pathomechanism associated with the usual fibrosis progression because of the absence of the conventional risk factors. It is expected that this will be clarified using genetic analysis and other methods in the future. Further to this point, the incidence of MPM-related HCC in non-fibrotic liver in a series can give us important hints about how we care for postoperative patients without known risk factors for HCC.

Finally, we must emphasize again the future role of genetic approaches. During the last 10 years, our understanding of molecular markers and genetic testing has remarkably improved, including Inc RNAs, miRNAs, and mRNAs [104,105]. Although these molecular or genetic tests were not performed in reported patients with HCC in non-fibrotic liver, these tests are expected to provide novel insights into the pathomechanism of HCC in nonfibrotic liver.

## 11. Conclusions

As discussed in our review, recent reports have slightly expanded our knowledge of HCCs in non-fibrotic livers. A large-scale Japanese survey shows a rising incidence of HCC in non-fibrotic livers and suggests that in the coming years, the incidence of HCC in non-fibrotic livers will continue to increase. Our review notes some clinical and tumor characteristics: (a) occurring mainly in young female and elder male patients, (b) a clinical lack of surveillance and lack of risk factors, (c) associated with metabolic disease, (d) macroscopically, a single and large lesion, and (e) microscopically well-differentiated and encapsulated. The peri-and post-operative complications were very few, but tumor recurrence appeared frequently despite R0 resection, thus, a close postoperative surveillance is needed.

We expect that this review not only fulfils our curiosity academically, but more importantly, helps to improve the clinical management of HCCs in non-fibrotic livers. Most clinicians believe that the occurrence of HCC is unlikely if their patients have no symptoms and their hepatic function is normal. The correction of this erroneous recognition is the first step for the appropriate management of HCC in normal patients.

However, this point of view might be affected due to limitations from an insufficient number of reported cases. Thus, more research is needed to verify it and explore the mechanisms involved.

Finally, we summarize two key takeaways from this review: (a) when caring for patients with normal hepatic function and without hepatic viral infection, we must not forget the possibility of HCC in non-fibrotic liver, and (b) the arterial hyperenhancement of the lesion and the presence of a capsule increase the possibility of HCC in non-fibrotic liver.

## Figures and Tables

**Table 1:** Summary of reported cases of HCC in non-fibrotic liver (1).

Year	Author [Reference]	Patient No.	Sex (M/F)	Age, Mean Age (Range)	HBV Infection	HCV Infection	AFP Normal/Elevated	Liver Enzyme Normal/Elevated	Metabolic Disease
2006	Eguchi [38]	27	16/11	59 (26–76)	0	0	ND	ND	ND
2007	Bège [39]	67	46/21	57	12	3	35/32	ND	ND
2008	Guzman [40]	3	1/2	57 (45–70)	0	0	2/1 (1533 ng/mL)	2/1	2/3
2008	Takahashi [41]	1	F	36	−	−	Normal	Normal	−
2013	Lewis [42]	1	F	75	−	+	Normal	ND	ND
2015	Komiyama [43]	1	M	76	−	−	Normal	Normal	+
2015	Sheng [44]	10	9/1	65 (4–8)	ND	ND	5/5	ND	ND
2019	Naganuma [45]	12	8/4	70.7 (50–86)	1	0	11/1 (1306 ng/mL)	7/5	3/12
2020	Ogasawara [46]	1	F	48	−	−	Elevated (224 ng/mL)	Normal	−
2022	Onishi [47]	1	F	23	−	−	Elevated (941.8 ng/mL)	Normal	−

Patient No.: patient number; M: male; F: female; HBV: hepatitis B virus; HCV: hepatitis C virus; AFP: α-fetoprotein; ND: no details given; −: negative; +: positive.

**Table 2:** Summary of reported cases of HCC in non-fibrotic liver (2).

Year	Author [Reference]	Patient No.	Tumor			Tumor Vascularity		Resection (Cases)
			Size (Range) (mm)	Histology wel/mod/por	Capsule (Cases)	Arterial Enhancement	Wash-Out	
2006	Eguchi [38]	27	90 (17–250)	19 (wel-mod)/2 (por) (6 unknown)	20/27	ND	ND	27
2007	Bège [39]	67	115	non-WHO	54/67	ND	ND	67
2008	Guzman [40]	3	71 (68–100)	1/2/0	ND	ND	ND	3
2008	Takahashi [41]	1	60	mod	+	hyper	+	1
2013	Lewis [42]	1	38	mod	ND	hyper	+	1
2015	Komiyama [43]	1	58	wel	+	hypo	ND	1
2015	Sheng [44]	10	<20	0/9/1	8/10	hyper 10/10	4/10	10
2019	Naganuma [45]	12	78.6 (23–200)	6/6/0	11/12	hyper 12/12	12/12	12
2020	Ogasawara [46]	1	25	por	−	hyper	+	1
2022	Onishi [47]	1	50	por	+	hyper	+	1

Patient No.: patient number; ND: no details given; −: negative; +: positive; hyper: hypervascular enhancement; hypo: hypovascular.

## Data Availability

Not applicable.
